# Piezogenic Pedal Papules*

**DOI:** 10.1590/abd1806-4841.20154884

**Published:** 2015

**Authors:** Bruno de Oliveira Rocha, Juliana Dumêt Fernandes, Fernanda Ventin de Oliveira Prates

**Affiliations:** 1 Universidade Federal da Bahia (UFBA) – Salvador (BA), Brazil.

**Figure 1:**
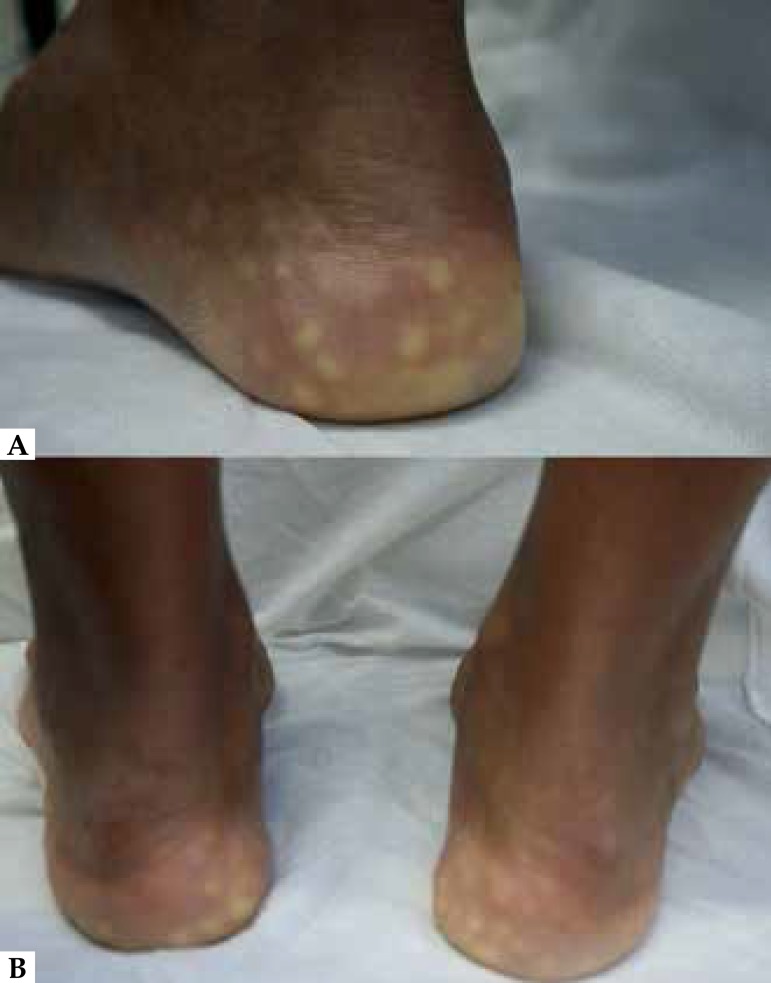
(A) Yellowish to skin-colored papules in the heels. (B) Symmetrical lesions

**Figure 2:**
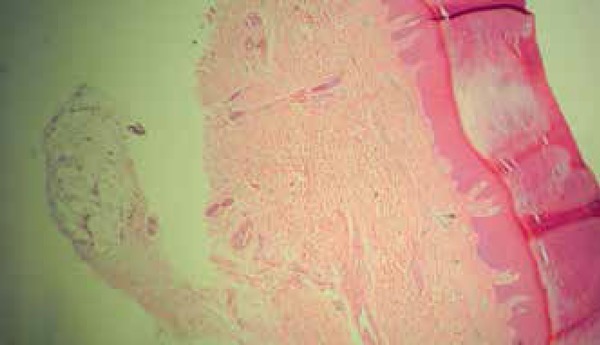
Thickened stratum corneum (Hematoxylin and eosin stain; original magnification X100)

Dear Editor,

A 24-years-old female sought medical assistance with complaints of a two-year history of bumps on her heels, initially asymptomatic, getting intensely painful over the time. She was generally healthy, and practiced running regularly. Physical examination revealed multiple skin-colored papules on both heels, more evident under pressure (Figure 1). Histopathological exam showed thickening of the stratum corneum (Figure 2). The diagnosis of painful piezogenic pedal papules (PPPP) was made.

PPPP were first described by Shelley and Rawnsley (1968).^1,2,3^ As the name "piezogenic" suggests, lesions are generated caused by pressure ("piesis"), inducing herniation of footpad's fat through the dermis.^1-4^ Constitutive fragility of the connective tissue may permit penetration of fat into upper layers of the skin, what would explain its association with congenital defects of the collagen, such as Ehlers-Danlos syndrome (EDS).^1-5^

Our patient presented with typical lesions: soft skin-colored to yellowish papules, located on the side of the heel, which became more apparent if subjected to pressure.^1,3,4^ There is description of a higher occurrence of piezogenic papules in runners (as in the current case), triathletes and individuals exposed to long periods of standing due to the greater time that soles are exposed to pressure.^1^ In a population-based study, the prevalence of piezogenic papules was found to be 2.5%, and 100% of these cases were associated with intense physical activity.^2^ Some cases have a familial pattern, and others occur simultaneously to some diseases such as Prader-Willi syndrome and, as pointed out earlier, EDS.^1,3-5^

Piezogenic papules are known to be common in healthy subjects, and the great majority experiences no symptom (90%).^1,3^ The pain in the remaining patients is resulting from ischemia of blood vessels and associated nerves, with consequent thickness of papillary dermis.^1-4^ Pain when standing is the main cause for seeking medical care.^1^

In the physical examination, the clinician can observe the protrusion of the fat lobules with the application of pressure on the feet.^1-4^ This handling not only reveals the fat pad protrusion, but also may reproduce the pain.^2^ Although rarely performed, biopsy specimens demonstrate thickened dermis, loss of the typical compartmentalization of the fat lobules, and thinning trabeculae in the subcutaneous fat.^1-3^ In our patient, biopsy showed only thickened stratum corneum (hyperkeratosis). Nevertheless, although histological findings corroborate the diagnosis, they are not a diagnostic criterion.^1^ In our case, the histopathology could be explained by lesions topography, and a possible superficiality of the sample. Then, facing a clinically typical case, we considered unnecessary to refer the patient to another biopsy.

The response to the treatments available for PPPP is often unsatisfactory.^2^ The decrease of time standing and weight loss can reduce symptoms.^1^ Compression hosiery, electroacupuncture, intralesional corticosteroid infiltration, and anesthetics have also been reported to reduce pain.^1-4^ If conservative measures fail, surgical removal may be an option.^1^ Our patient has undergone electroacupuncture sessions previously, without success, and, at our service, we performed corticosteroids intralesional infiltration, with pain relief.

The diagnosis of PPPP can be a challenge for professionals who are unaware to this entity, as patients may present with an apparently normal physical examination.
